# A Non–Gas-Based Cryotherapy System for the Treatment of Cervical Intraepithelial Neoplasia: A Mixed-Methods Approach for Initial Development and Testing

**DOI:** 10.9745/GHSP-D-16-00270

**Published:** 2017-03-24

**Authors:** Miriam Cremer, Proma Paul, Katie Bergman, Michael Haas, Mauricio Maza, Albert Zevallos, Miguel Ossandon, Jillian D Garai, Jennifer L Winkler

**Affiliations:** a Obstetrics, Gynecology & Women's Health Institute, Cleveland Clinic Lerner College of Medicine, Cleveland, OH, USA.; b Basic Health International, New York, NY, USA, and San Salvador, El Salvador.; c University of Pittsburgh Graduate School of Public Health, Pittsburgh, PA, USA.; d CryoPen, Inc., Covington, LA, USA.; e Department of Family Medicine, Louisiana State University Health Science Center, New Orleans, LA, USA.; f Department of Gynecology, National Institute for Neoplastic Diseases, Lima, Peru.; g National Institutes of Health, U.S. Department of Health and Human Services, Bethesda, MD, USA.; h Mel & Enid Zuckerman College of Public Health, University of Arizona, Tucson, AZ, USA.

## Abstract

**Background:**

Gas-based cryotherapy is the most widely used treatment strategy for cervical intraepithelial neoplasia (CIN) in low-resource settings, but reliance on gas presents challenges in low- and middle-income countries (LMICs). Our team adapted the original CryoPen Cryosurgical System, a cryotherapy device that does not require compressed gas and is powered by electricity, for use in LMICs.

**Methods:**

A mixed-methods approach was used involving both qualitative and quantitative methods. First, we used a user-centered design approach to identify priority features of the adapted device. U.S.-based and global potential users of the adapted CryoPen participated in discussion groups and a card sorting activity to rank 7 features of the adapted CryoPen: cost, durability, efficacy and safety, maintenance, no need for electricity, patient throughput, and portability. Mean and median rankings, overall rankings, and summary rankings by discussion group were generated. In addition, results of several quantitative tests were analyzed including bench testing to determine tip temperature and heat extraction capabilities; a pathology review of CIN grade 3 cases (N=107) to determine target depth of necrosis needed to achieve high efficacy; and a pilot study (N=5) investigating depth of necrosis achieved with the adapted device to assess efficacy.

**Results:**

Discussion groups revealed 4 priority themes for device development in addition to the need to ensure high efficacy and safety and low cost: improved portability, durability, ease of use, and potential for cure. Adaptions to the original CryoPen system included a single-core, single-tip model; rugged carrying case; custom circuit to allow car battery charging; and sterilization by high-level disinfection. In bench testing, there were no significant differences in tip temperature or heat extraction capability between the adapted CryoPen and the standard cryotherapy device. In 80% of the cases in the pilot study, the adapted CryoPen achieved the target depth of necrosis 3.5 mm established in the pathology review.

**Conclusion:**

The LMIC-adapted CryoPen overcomes barriers to standard gas-based cryotherapy by eliminating dependency on gas, increasing portability, and ensuring consistent freeze temperatures. Further testing and evaluation of the adapted CryoPen will be pursued to assess scalability and potential impact of this device in decreasing the cervical cancer burden in LMICs.

## Abstract (precis)

A non–gas-based treatment device for early cervical cancer treatment, adapted for use in low-resource settings to improve ease of use, portability, and durability, performed similarly to a standard gas-based cryotherapy device in small-scale testing. A large randomized clinical trial is currently underway for further assessment.

## BACKGROUND

Cervical cancer continues to be a global public health problem, with the greatest disease burden in low- and middle-income countries (LMICs).^1^ The number of women screened for cervical cancer has increased in many countries over the past decade.^2^ However, screening must be linked with effective, affordable treatment in order to achieve a reduction in cervical cancer incidence and mortality.

Gas-based cryotherapy is the most widely used treatment strategy for cervical intraepithelial neoplasia (CIN) in low-resource settings. This ablative technique uses compressed carbon dioxide or nitrous oxide gas to freeze lesions, resulting in necrosis of cells. Although gas-based cryotherapy is safe, simple, and generally inexpensive, challenges include difficulties with procuring gas, transporting and refilling gas tanks, and providing treatment in high-volume settings.^3^^–^^5^ A standard-size gas tank has the capacity to provide treatment for only 3 patients, while a large tank can treat more women but is difficult to transport from clinic to clinic.^6^

Gas-based cryotherapy is the most widely used treatment for cervical neoplasia in low-resource settings, but challenges around procuring gas and transporting and refilling gas tanks exist.

Innovative new technologies offer treatment alternatives. For example, the CryoPen Cryosurgical System is a novel cryotherapy technology that does not require compressed gas and is powered by electricity. The CryoPen reaches extreme cold temperatures with use of a Stirling Cryocooler that chills the core to operational temperatures of −105°C. Between treatments, the core rests in the chilling well filled with an ounce of ethanol or grain alcohol, which facilitates heat transfer. The CryoPen was originally designed to treat dermatologic conditions and was modified for gynecologic use in 2011. It is currently approved by the U.S. Food and Drug Administration (FDA) for CIN grade 2 and more severe (CIN2+) diagnoses.

The CryoPen Cryosurgical System, which does not require compressed gas, was originally designed to treat dermatologic conditions and was later modified for gynecologic use.

The original CryoPen device, however, is not conducive for use in LMICs. Our team of clinicians and biomedical engineers collaborated to develop an LMIC-adapted CryoPen optimized for use in low-resource settings. To design the LMIC-adapted CryoPen prototype, we sought guidance on features that would meet the needs of clinicians interested in using this technology in LMICs. We followed a user-centered design approach to better understand current cryotherapy methods in LMICs, features that would be essential in an LMIC-adapted CryoPen, advantages and disadvantages of the initial prototype, and factors that may facilitate adoption of the adapted device.

We adapted the CryoPen system for use in low-resource settings following a user-centered design approach to identify essential features.

To ensure that the LMIC-adapted CryoPen is non–inferior to standard cryotherapy devices, we performed in vitro studies using ballistic gelatin and the double-freeze approach recommended by the World Health Organization to determine the prototype's tip temperature and heat extraction capabilities.^7^ A pilot study was performed using an exploratory single-freeze approach to compare the depth of necrosis achieved by the LMIC-adapted CryoPen and CO_2_-based cryotherapy in healthy cervical tissue.

The purpose of this article is to describe the design approach and device adaptations of the LMIC-adapted CryoPen and to report initial clinical data.

## MATERIALS AND METHODS

### Qualitative Methods: Experiences With Cryotherapy and Device Perceptions

Using a convenience sample, we held 3 focus group discussion sessions with key stakeholders and potential users of the LMIC-adapted CryoPen. Verbal consent to participate was obtained prior to the discussions. None of the participants were compensated for their participation in any way.

During the first session, at CryoPen, Inc. headquarters in September 2014, 10 cervical cancer prevention stakeholders met to discuss the development of the LMIC-adapted CryoPen. Two additional discussion sessions were held in conjunction with the Global Academic Partnership (GAP) conference in Houston, Texas, in April 2015. Most of the stakeholders who participated in the September discussion session participated in the second session. From the larger group of GAP conference attendees, we identified participants who worked in cervical cancer prevention globally and invited them to join the third discussion session. This third group consisted of 22 potential users or key stakeholders from Brazil, China, Colombia, India, Mexico, South Africa, Thailand, and Zambia, as well as U.S. participants who have performed cryotherapy in Guatemala and Peru. The stakeholders were all experienced in cervical cancer prevention and included trainers or practitioners in visual inspection with acetic acid (VIA) and cryotherapy, a cryotherapist, a colposcopist, gynecologic oncologists, a pathologist, a radiologist, and a registry and cervical cancer prevention program planner.

During the second and third discussion sessions, the participants used a card sorting activity to rank 7 features of the LMIC-adapted CryoPen and then discussed the potential design trade-offs in various settings. Participants described their experiences with cryotherapy and shared feedback on the LMIC-adapted CryoPen prototype.

Digital recordings and field notes were reviewed using an iterative content analysis approach for qualitative analysis to identify emerging themes. Key quotes were transcribed to illustrate identified themes. We calculated a mean and median ranking for each feature included in the card sorting exercise and generated an overall ranking for each feature as well as a summary ranking for each by discussion group.

The University of Pittsburgh Institutional Review Board (IRB) determined that the focus group discussions qualified as non–research.

### Quantitative Methods: Device Testing

#### Bench Testing

Bench testing of the LMIC-adapted CryoPen and a standard cryotherapy device, the N_2_O-based Wallach LL100 System, was performed in the Basic Health International laboratory in San Salvador, El Salvador. The office lab has the capacity to maintain consistent room temperature and humidity, which provided controlled testing between trials. No animals or hazardous materials were used in testing. An engineer who graduated from the ABET-accredited bioengineering program at Rice University conducted the bench tests.

For comparative studies of the tip temperature and heat extraction capabilities of the 2 devices, ballistic gelatin was used as the cervical tissue analogue. It is not a perfect surrogate, but the bias is the same for all devices that were tested. Ballistic gelatin, composed of gelatin powder and water, is FDA-cleared to test gynecologic ablation devices such as the Her Option Uterine Cryoblation Therapy System (PMA Number P000032). Standard procedure was followed for preparing ballistic gelatin samples for testing. The starting temperature of the gelatin samples was room temperature. A custom jig was designed to perform freezing tests on ballistic gelatin using the CryoPen Cryosurgical System (both standard and prototype LMIC-adapted models). Fifteen trials were conducted with each device using a double-freeze approach consisting of a 3-minute freeze, followed by a 5-minute thaw, and a second 3-minute freeze (3'–5'–3'), which is the recommended treatment approach per the most recent World Health Organization guidelines for cryotherapy.^7^

Heat extraction capabilities were measured in terms of mass of the freeze ball and the lateral freeze and depth of freeze dimensions. At the completion of the 3'–5'–3' freeze cycle, the freeze ball was excised from the gelatin. Excess gelatin was removed from the freeze ball before the mass was recorded on a scale. After the freeze ball mass was recorded, the lateral freeze and depth of freeze dimensions were recorded using calipers. Fifteen tests of each set of data points were performed to ensure data collection replication. Copper-constantan thermocouples and an Omega Instruments Data Acquisition system were used to collect temperature data.^8^

#### Pathology Review to Determine Target Depth of Necrosis

We investigated the depth of CIN in order to determine target depth of necrosis required for high efficacy of the device. A study of the depth of CIN1–3 by Abdul-Karim showed a depth of necrosis of 3.5 mm is needed to treat 95% of CIN1–3 lesions and a depth of 4.8 mm is needed to treat 99% of all cases.^9^ With our collaborators at the National Institute for Neoplastic Diseases (INEN) in Peru, our group analyzed 107 confirmed cases of CIN3 in cold knife cone biopsy specimens from an under-screened population. Two expert pathologists reviewed slides of previously confirmed cases of CIN3. INEN and Cleveland Clinic granted IRB approval.

Depth of CIN3 ranged from 0.2 mm to 6.9 mm, with a mean of 2.0 mm. Overall, 85/107 (79.4%) had a depth <3.0 mm, 96/107 (89.7%) had a depth <3.5 mm, 100/107 (93.5%) had a depth <4.0 mm, and 100/107 (93.5%) had a depth <5.0 mm. Using these findings and Abdul-Karim's conclusion that a depth of 3.5 mm is needed to treat 95% of CIN2+ lesions, we determined that our clinical goal was to achieve a minimum depth of necrosis of 3.5 mm in at least 80% of cases. Since cryotherapy is approximately 80% effective in treating CIN, we set 80% as the goal in order to determine equivalence.

Our clinical goal was to achieve a minimum depth of necrosis of 3.5 mm in at least 80% of cases in order to determine equivalence to standard cryotherapy treatment.

#### Pilot Study to Determine Depth of Necrosis Achieved With LMIC-Adapted CryoPen

A pilot study (N=5) was then conducted to determine if the LMIC-adapted CryoPen achieved a depth of necrosis of 3.5 mm in women with healthy cervical tissue. Women aged 21–64 years who presented at INEN for hysterectomy indicated by conditions unrelated to cervical precancer or cervical cancer were invited to participate. Women did not receive any financial incentive to participate. IRB approval for the pilot study was granted by both INEN and Cleveland Clinic.

Five women were treated with a single 5-minute (5') freeze application of the LMIC-adapted CryoPen; the straightforward 5' freeze was chosen so that providers could become comfortable with the device. The participants then underwent their prescheduled hysterectomy 24 hours after treatment, and cervical tissue was processed to allow evaluation of depth of necrosis caused by ablation. The entire cervix obtained from the hysterectomy procedures was detached from the uterus and cut at 3 and 9 o'clock positions to separate the anterior and posterior lips. Both pieces of tissue were fixed in formalin for 24 hours. Multiple serial sections of specimens were obtained from each of the cervical lips.

Five women prescheduled for hysterectomy for reasons unrelated to cervical precancer or cancer were first treated with the adapted CryoPen to determine depth of necrosis achieved.

The pathologists evaluating the cervical specimens were blinded to which device was used to perform the ablative treatment. Microscopic evaluation of depth of necrosis was conducted by superimposing a micrometer in the 10x eyepiece. Multiple measurements were taken and only the deepest area of necrosis was recorded for this study. Determination of the deepest level of necrosis was based on the observation of destruction of the glandular epithelium in the gland crypts in the stroma or of the endothelium of the stromal blood vessels.

## RESULTS

### Qualitative Results

#### Experience With Cryotherapy

Providers in our group with experience in Colombia, Guatemala, Peru, Thailand, and Zambia described the context in which they use cryotherapy for precancerous cervical lesions. Cryotherapy is practiced broadly in Thailand and is provided either at a health center or through a mobile clinic. In Zambia, cryotherapy is performed at regional health facilities; although the mobile units maintained by the Ministry of Health could provide cryotherapy, this is not currently practiced. In Colombia, precancerous cervical lesions are primarily treated through excision techniques; cryotherapy is performed only in remote counties. Other providers in our group from Brazil, China, and India reported using only excisional techniques such as loop electrosurgical excision procedure (LEEP) and had limited experience with cryotherapy.

#### Device Perceptions

The card sorting activity facilitated the core conversation in which participants ranked the most important features (out of 7) for a new cryotherapy device designed for the low-resource settings in which they practice. The 7 features and their overall rankings are as follows:

**Efficacy and safety (overall ranking 1)**: While there was some variation in the ranking of efficacy and safety, this feature was ranked highest overall. A participant from Zambia noted:

Efficacy and safety was the most important [feature] because whatever new device goes into the market, it has to be non–inferior to the current practice.

Card sorting participants ranked efficacy and safety highest in terms of desired features of an adapted CryoPen.

**Cost (overall ranking 2)**: Cost was ranked either very high or very low, but the summary ranking was second overall. Participants felt that the target price of US$4,000 for the new device was reasonable, especially given that this device would not require purchase of gas.

**Durability and Maintenance (overall ranking 3):** Durability and maintenance, 2 separate features, were discussed in interwoven language. Durability was considered a core issue for this setting. Participants defined durability in terms of simplicity and the concept that a device could not be broken. A participant from the United States who has practiced broadly in low-resource settings said:

I ranked durability first, and part of that is that I feel like every public hospital I've gone to in low- and middle-income countries, you can barely walk down the hall because there are all these donated instruments from the U.S. lining the halls that don't work, that are broken … You know, no one knows how to maintain them.

Another participant who has practiced cryotherapy in Guatemala raised the need for simplicity of design:

… but as you've shown you have the [thermo]coupler, a microchip, a something, it sounds like there are a lot more things that could break there than your typical cryo unit and a nitrous tank. … ‘cause the only thing that can go wrong with the [traditional] cryo gun is if you try to use it when the pressure is too high in the tank.

Participants were supportive of simple inexpensive maintenance, but reiterated the importance of durability. Participants also acknowledged that routine maintenance has generally not been practiced with current cryotherapy equipment.

**Portability (overall ranking 4):** Portability was discussed in terms of the ability to bring a device to a remote setting. Participants felt that a truly portable device would open up the option of delivering cryotherapy in remote settings that are not currently being reached because of the logistical difficulties of transporting the gas tank needed for standard cryotherapy. A participant from Zambia noted:

If it is not portable then it can't get to the rural areas where you want to reach out.

**Patient throughput (overall ranking 5)**: Participants explained that the proposed target of each device being able to treat 15 patients per day was acceptable, and so they saw no need to prioritize this issue above others. One participant explained:

When we screen about 200–250 [patients], we only freeze about 15 a day, that is why it was at the bottom.

**No electricity (overall ranking 6)**: Many sites where cryotherapy will be practiced have electricity. Participants felt that the proposed approach of paying an additional cost for a modularized additional battery feature would adequately address the need to bring the device to remote areas that do not have electricity.

#### Integrating Feedback to Optimize the LMIC-Adapted CryoPen Cryosurgical System

The device prototype was modified based on the 4 priority areas that came out of the focus group discussions: (1) improved portability—essential for large mobile health services that many countries rely on to treat women living in remote areas, (2) better durability, which allows for decreased maintenance and costs, and increases capacity for rugged travel and use, (3) greater ease-of-use, which simplifies operation of the device and expands potential for use by trained providers, and (4) enhanced potential for cure, which ensures greater efficacy for the treatment of precancerous lesions.

**FIGURE 1:**
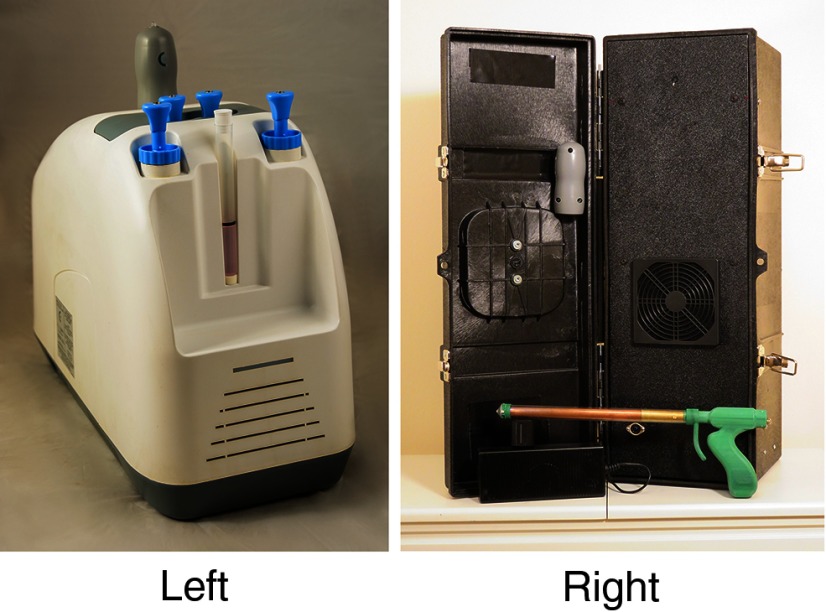
Standard CryoPen System Available in the United States (left) and the CryoPen System Adapted for Use in Low- and Middle-Income Countries (right)

The LMIC-adapted CryoPen Cryosurgical System has several design modifications that distinguish it from the original CryoPen (Figure 1). To increase efficacy of treatment, a single 19–20 mm conical tip was designed for the new model. Compared with the original model's tip, the new larger tip size ensures that the adapted device achieves greater coverage of precancerous lesions, and the anatomically correct tip shape allows for better contact with the cervix. The original CryoPen required a core swap mid-procedure, whereas the LMIC-adapted CryoPen has a single core that can be applied for a 3'–5'–3' or a single 5' procedure. A clear sheath, made of medical-grade polycarbonate, increases visibility and protects the vaginal walls. The sheath is inserted with the attached tip positioned against the cervix; the core is then inserted into the sheath, with a gap preventing contact between the sheath and core for insulation purposes.

To increase durability, the new model was built to handle irregular currents and to withstand physical damage in extreme conditions (ambient temperatures of −100°C to 40°C) and from drops up to 5 feet. In addition, the new model is easily maintainable. The device has a removable air filter, which can be removed, cleaned, and replaced on site. The protective sheath and attached tip are the only components in direct contact with human tissue and can be cleaned sufficiently with high-level disinfection (HLD) rather than requiring autoclave sterilization.

The LMIC-adapted device is portable, weighing 20 lbs., and equipped with a handle. To address focus group participants' interest in a modularized approach to a battery feature, a custom circuit was designed to allow the LMIC-adapted CryoPen to run via car batteries for use in settings without another source of electricity.

The adapted CryoPen is portable and can run on car batteries in settings without another source of electricity.

### Quantitative Results: Device Testing

#### Results of Bench Testing

The results of the 15 bench test trials showed that the LMIC-adapted CryoPen achieved equivalent depth of freeze, depth of lateral freeze, diameter of freeze, and mass of freeze ball compared with the Wallach LL100 (Table 1). The minimum tip temperatures reached by both devices and the average of the temperatures reached during the 2 freezes were also equivalent. There were no significant differences between devices in any tip temperature or heat extraction capability measurements.

**TABLE 1.** Bench Testing Results Comparing Heat Extraction Capabilities and Tip Temperatures of Standard Cryotherapy (Wallach LL100) Versus LMIC-Adapted CryoPen

	Wallach LL100 (n=15) Mean (SD)	LMIC-Adapted CryoPen (n=15) Mean (SD)	*P* Value
Depth of freeze, mm	7.14 (1.00)	6.33 (1.21)	.06
Lateral depth of freeze, mm	7.86 (0.96)	7.93 (1.32)	.88
Diameter of freeze, mm	29.37 (2.09)	30.71 (3.61)	.23
Mass of freeze ball, g	7.71 (1.16)	8.27 (1.33)	.22
Average tip temperature in freeze cycle, °C	−51.84 (4.99)	−48.28 (6.37)	.10
Minimum (coldest) tip temperature, °C	−56.11 (4.19)	−55.08 (7.42)	.64

#### Depth of Necrosis Pilot Study

Average maximum depth of necrosis in the anterior lip was 4.12 mm (standard deviation [SD], 1.49 mm), and average maximum depth in the posterior lip was 4.08 mm (SD, 1.05 mm) (Table 2). A depth of necrosis of 3.5 mm was achieved in 80% of the samples (maximum depth of necrosis was ≥4.0 mm in 80% of cases).

**TABLE 2.** Preliminary LMIC-Adapted CryoPen Depth of Necrosis with a Single 5-Minute Freeze

Sample	Maximum Anterior Depth of Necrosis (mm)	Maximum Posterior Depth of Necrosis (mm)
1	4.0	3.9
2	1.5	2.8
3	6.1	6.0
4	4.5	3.8
5	4.5	3.9
Mean	4.12	4.08

Bench trial tests showed the adapted CryoPen performed equivalently to the standard cryotherapy device, and the pilot test found depth of necrosis of at least 3.5 mm was achieved in 80% of the samples.

## DISCUSSION

Despite the continued need for secondary prevention of cervical cancer via treatment of precancerous lesions, there have been few efforts to develop treatment devices tailored to the specific challenges and needs of LMICs. In developing the LMIC-adapted CryoPen cryotherapy treatment device, each adaptation was made specifically to optimize utility in low-resource settings while maintaining high efficacy and safety. The user-centered design approach used in this study was an effective way to solicit input about important features to potential users and helped inform development of the CryoPen technology for LMIC settings.

Participants were enthusiastic about the potential of a non–gas-based cryotherapy device. The LMIC-adapted CryoPen overcomes barriers to standard gas-based cryotherapy by eliminating dependency on gas, increasing mobility, and ensuring consistent freeze temperatures without blockages; the device can perform 3 procedures per hour when connected to an electrical source. Participants emphasized that durability and simplicity were important factors, highlighting the need for the new device to survive being moved frequently from one location to another in hot environments without requiring intensive maintenance. Product life, which remains unknown despite promising durability, was therefore also an outstanding concern. With these concerns in mind, the LMIC-adapted CryoPen was designed with intention to enhance 3 key features:

1. **Durability/Maintenance.** Features include the ability to handle irregular currents; withstand physical damage in extreme temperatures; tolerate drops from 5 feet; be cleaned by HLD; and be maintained easily with a removable air filter.
2. **Portability.** Features include a lightweight device; a carrying case with handle; and the ability to run on 2 car batteries in series when electricity is unavailable.
3. **Ease-of-use.** Features include a single-tip, single-core device; a clear sheath for increased visibility and safety; and a device amenable to one-handed operation.

The adapted CryoPen device overcomes barriers to standard gas-based cryotherapy by eliminating the need for gas and increasing mobility.

Safety and efficacy were the most important features to participants, as introducing this technology to the current treatment paradigm will require non–inferiority to current practice. We ensured non–inferiority in safety and efficacy to the current standard, gas-based cryotherapy, by conducting bench testing of the LMIC-adapted CryoPen and through a clinical trial pilot determining the depth of necrosis the device achieved in healthy cervical tissue. The average tip temperature of both freezes (−51.84°C) reached by the device is in a range theoretically capable of destroying cervical tissue.^10^^–^^12^ The LMIC-adapted CryoPen achieved equivalent heat extraction capabilities, including depth of freeze, lateral depth of freeze, and mass of freeze ball, compared with the standard Wallach LL100. During the clinical trial pilot, the LMIC-adapted CryoPen achieved a depth of necrosis of at least 3.5 mm in 80% of cases, demonstrating the promising efficacy of the device. Given the consistent performance in bench testing, the freeze that performed less well in the clinical trial pilot may be attributable to the process of clinicians adjusting to the new technology in a clinical setting. The sample size of the pilot study was small. A large randomized clinical trial that will more fully investigate safety and efficacy issues is currently underway; the results of this trial will have greater precision than the pilot.

A randomized clinical trial is underway to more fully investigate safety and efficacy issues.

Despite the advantages presented by the LMIC-adapted CryoPen, there are several limitations associated with the device. The $4,000 cost, although within the range deemed reasonable by focus group participants, is more expensive than a gas-based system ($2,000). This is a significant investment for areas with limited funding. However, the initial capital cost of the device is mitigated by the elimination of ongoing gas expenses for clinics. Depending on frequency of use, the cost difference between the LMIC-adapted CryoPen and a gas-based system would be negligible within 6–18 months of use.

The initial capital cost of the adapted CryoPen is offset by eliminating the need for ongoing gas expenses.

We recognize that costs are entailed by the device's electricity requirement, but we believe that even with this constraint the LMIC-adapted CryoPen will be more affordable than conventional cryotherapy. In cost-effectiveness analysis, a health economist will account for both sources of electricity that can be utilized by the device—alternating current supplied by an electrical grid and car batteries.

The LMIC-adapted CryoPen was designed to be simple and user-friendly, but there are steps that must be followed during use. These include filling the chilling well with ethanol; ensuring that the green indicator light is on before beginning treatment; and wiping condensation from the core before reinserting it into the holding well. These steps are simple, but they are unique to this device and essential for proper functioning. As with introducing any new device, proper training and repeated use will address these concerns. An instruction booklet and video are available for reference.

Although the device does not require N_2_O or CO_2_, ethanol is needed to prevent the core from freezing to the well in which it rests between procedures. Manufacturer-approved ethanol is relatively easy to find and inexpensive; if unavailable, ethanol can be substituted with grain alcohol such as Everclear. A small bottle can facilitate hundreds of treatments. It will nonetheless be important that procurement systems include ethanol for use with this device.

Further testing and evaluation of the LMIC-adapted CryoPen should be pursued to assess scalability and potential impact of this device. Despite introduction of the prophylactic human papillomavirus (HPV) vaccine, generations of unvaccinated women will still need to be screened and treated for cervical precancer. In the 3 decades it may take to implement primary prevention of cervical cancer by HPV vaccine globally, an estimated 20 million more women will be diagnosed with cervical cancer in LMICs.^13^ If improvements in secondary prevention—including greater utility of treatment devices in low-resource areas—are not pursued, more than 250,000 women will continue to die annually from a preventable disease.

## Notes

**Peer Reviewed**
